# Supplementary material for: DPP9: Comprehensive In Silico Analyses of Loss of Function Gene Variants and Associated Gene Expression Signatures in Human Hepatocellular Carcinoma
Source: Cancers (Basel). 2021 Apr 1;13(7):1637. doi: 10.3390/cancers13071637 (PMC8037973; doi:10.3390/cancers13071637)
Supplement: Supplementary file 1 [file cancers-13-01637-s001.pdf]

Supplementary Table 1. *DPP8* LoF variants in TCGA. Simple somatic mutation (SSM) affected frequency is calculated as the number of cases affected by a specific mutation in a TCGA disease project divided by the number of cases tested for SSM in that disease project in TCGA. ins = insertion. del = deletion. * Premature termination codon (PTC).

Position	Reference	Alternate	Protein consequence	Annotation	Case ID	Disease	Number of SSM affected cases and frequency
65479016delA			p.Gln458Lys*29	Frameshift	TCGA-AZ-6598<br>TCGA-AD-5900	Colon Adenocarcinoma	2/400 (0.50%)
65479011_65479012insG			p.Gln458Pro*8	Frameshift	TCGA-HU-A4GT	Stomach Adenocarcinoma	1/440 (0.23%)
65474260delC			p.Ile512*	Frameshift	TCGA-12-0778	Glioblastoma Multiforme	1/393 (0.25%)
65467180_65467181insAGGTAAATTATTAGTCAATT			p.Thr543Lys*13	Frameshift	TCGA-13-1500	Ovarian Serous Cystadenocarcinoma	1/463 (0.23%)
65456248_65456249insAAATTTAAGCCCTCGGTGACAGGATCCCCTGTTGAGGGCTTAAATTTGAAGGCGCCTTT			p.Glu715Lys*31	Frameshift	TCGA-4V-A9QI	Thymoma	1/123 (0.81%)
65454301delAGAGGTATCC			p.Gly758Pro*2	Frameshift	TCGA-AP-A1DO	Uterine Corpus Endometrial Carcinoma	1/530 (0.19%)
65500743	C	A	p.Glu153*	Stop gained	TCGA-BK-A6W3	Uterine Corpus Endometrial Carcinoma	1/530 (0.19%)
65500731	C	A	p.Glu157*	Stop gained	TCGA-D1-A17Q	Uterine Corpus Endometrial Carcinoma	1/530 (0.19%)

65500639	A	T	p.Tyr187*	Stop gained	TCGA-VQ-A91D	Stomach Adenocarcinoma	1/440 (0.23%)
65490237	C	A	p.Glu276*	Stop gained	TCGA-AX-A05Z<br>TCGA-AJ-A5DW	Uterine Corpus Endometrial Carcinoma	2/530 (0.38%)
					TCGA-AA-3510	Colon Adenocarcinoma	1/400 (0.25%)
65480371	G	A	p.Gln399*	Stop gained	TCGA-D3-A8GK	Skin Cutaneous Melanoma	1/469 (0.21%)
65478907	G	A	p.Arg493*	Stop gained	TCGA-AG-A02N	Rectum Adenocarcinoma	1/137 (0.73%)
65467149	G	C	p.Tyr553*	Stop gained	TCGA-Q1-A6DW	Cervical Squamous Cell Carcinoma and Endocervical Adenocarcinoma	1/289 (0.35%)
65467135_65467136insTTGTCATCCACCTACCTCGG			p.Val558A*6	Stop gained	TCGA-24-1431	Ovarian Serous Cystadenocarcinoma	1/436 (0.23%)
65467124	C	A	p.Glu562*	Stop gained	TCGA-29-1768	Ovarian Serous Cystadenocarcinoma	1/436 (0.23%)
65456263	G	A	p.Arg710*	Stop gained	TCGA-AZ-4615	Colon Adenocarcinoma	1/400 (0.25%)
65454394	G	A	p.Gln730*	Stop gained	TCGA-ZP-A9CY	Liver Hepatocellular Carcinoma	1/364 (0.27%)

Supplementary Table 2. *DPP8* LoF variants in COSMIC. Nonsense mutation is a substitution mutation resulting in a premature termination codon (*). CDS = coding sequence; AA = amino acid, SSM = simple somatic mutation.

CDS mutation	AA mutation	Legacy mutation ID	Type	Disease	Number of SSM affected cases
c.2262G>A	p.Trp754*	COSM5946730	Nonsense	Lymphoid neoplasm	1
c.2017C>T	p.Gln673*	COSM6574976	Nonsense	ER-PR-positive breast carcinoma	1
c.1659C>G	p.Tyr553*	COSM4856031	Nonsense	Cervical squamous cell carcinoma	1
c.328G>T	p.Glu110*	COSM1678543	Nonsense	Colon adenocarcinoma	2
c.2128C>T	p.Arg710*	COSM3690497	Nonsense	Colon adenocarcinoma	1
c.826G>T	p.Glu276*	COSM964071	Nonsense	Endometrioid carcinoma Colon adenocarcinoma	3
c.1477C>T	p.Arg493*	COSM167000	Nonsense	Colon adenocarcinoma	1
c.2170C>T	p.Gln724*	COSM6648997	Nonsense	Colon adenocarcinoma	1
c.457G>T	p.Glu153*	COSM8970383	Nonsense	Endometrioid carcinoma	1
c.469G>T	p.Glu157*	COSM964073	Nonsense	Endometrioid carcinoma	1
c.2188C>T	p.Gln730*	COSM8423812	Nonsense	Hepatocellular carcinoma	1
c.2649C>G	p.Tyr883*	COSM88480	Nonsense	Ovarian clear cell carcinoma	1
c.1684G>T	p.Glu562*	COSM1323886	Nonsense	Ovarian serous carcinoma	1
c.1816G>T	p.Glu606*	COSM3981612	Nonsense	Ovarian mixed adeno-squamous carcinoma	1
c.2218C>T	p.Arg740*	COSM5929565	Nonsense	Skin basal cell carcinoma	1
c.1927G>T	p.Gly643*	COSM7894053	Nonsense	Malignant melanoma	1
c.1195C>T	p.Gln399*	COSM8050061	Nonsense	Malignant melanoma	1
c.1228G>T	p.Glu410*	COSM7945996	Nonsense	Malignant melanoma	1
c.2352G>A	p.Trp784*	COSM135677	Nonsense	Skin squamous cell carcinoma	1
c.561T>A	p.Tyr187*	COSM8209774	Nonsense	Stomach adenocarcinoma	1

Supplementary Table 3. Genome-wide significant loci for severe COVID-19: Intronic *DPP9* variants rs12610495 and rs2109069. hg19_coordinates: the hg19 chromosome position. Hg38_coordinates: the hg38 chromosome position. a1: the effect allele (aligned to the + strand). a2: the non-effect allele (aligned to the + strand). afr/amr/eas/eur/sas: the allele frequency for A1 in AFR/AMR/EAS/EUR/SAS population in 1000 Genomes. beta: association between the trait and the SNP expressed per additional copy of the effect allele (odds ratio is given on the log-scale). efo: the experimental factor oncology term for the phenotype or disease. AFR = African; AMR = American; EAS = East Asian; EUR = European; SAS = South Asian.

		Genomic location				Allele frequencies												
**Gene**	**rsid**	**hg19_coordinates**	**hg38_coordinates**	**a1**	**a2**	**afr**	**amr**	**eas**	**eur**	**sas**	**beta**	**Standard error of beta**	***p* value**	**Number of individuals**	**Dataset ID**	**Trait (phenotype or disease)**	***p* value COVID**	**efo**
DPP9	rs12610495	chr19:4717672	chr19:4717660	A	G	0.872	0.797	0.857	0.706	0.828	NA	NA	1.68E-12	47644	GRASP	Fibrotic idiopathic interstitial pneumonias pulmonary fibrosis	5.20E-06	NCIT_C35714
DPP9	rs12610495	chr19:4717672	chr19:4717660	A	G	0.872	0.797	0.857	0.706	0.828	-0.255	0.0362	2.00E-12	-	NHGRI-EBI_GWAS_Catalog	Interstitial lung disease	5.20E-06	EFO_0004244
DPP9	rs2109069	chr19:4719443	chr19:4719431	A	G	0.196	0.219	0.14	0.321	0.186	NA	NA	2.42E-11	47644	GRASP	Fibrotic idiopathic interstitial pneumonias pulmonary fibrosis	2.41E-05	NCIT_C35714

Supplementary Table 4. The LIHC/HCC (n = 360) and UCEC (n = 540) patient demographics

**Characteristic**	**LIHC/HCC Number and percentage or median and range ***	**UCEC Number and percentage or median and range ***
Sex		
Male	244 (67.8%)	/
Female	116 (32.2%)	540 (100%)
Age at diagnosis (years)	61 (16, 85)	64 (31, 90)
Tumour site		
Liver	360 (100%)	
Endometrium		525 (97.2%)
Fundus uteri		6 (1.11%)
Corpus uteri		4 (0.74%)
Isthmus uteri		3 (0.56%)
Overall death	126 (35%)	91 (16.9%)
Follow up time (days)	587 (1, 3675)	885 (0, 6859)
AJCC stage		
I	169 (46.9%)	334 (61.9%)
II	83 (23.1%)	52 (9.6%)
III	83 (23.1%)	123 (22.8%)
IV	4 (1.11%)	29 5.4%)

*Missing data excluded in percentage calculation

Supplementary Table 5. Cox proportional hazards model with gender as the covariate to evaluate associations between DPP9 expression and survival. HR = hazard ratio; CI = confidence interval.

**Variable**	**Multivariate cox P**	**HR (CI)**
DPP9	0.97	0.99 (0.69-1.41)
Gender	0.3	0.82 (0.57-1.19)

Supplementary Table 6. Cox proportional hazards model with BMI as the covariate to evaluate associations between DPP9 expression and survival. HR = hazard ratio; CI = confidence interval.

**Variable**	**Multivariate cox P**	**HR (CI)**
DPP9	0.55	1.54 (0.71-3.35)
Overweight	0.7	0.96 (0.8-1.15)
DPP9	0.27	0.75 (0.3-1.89)
Obese/Extreme obese	0.98	0.99 (0.7-1.29)

A

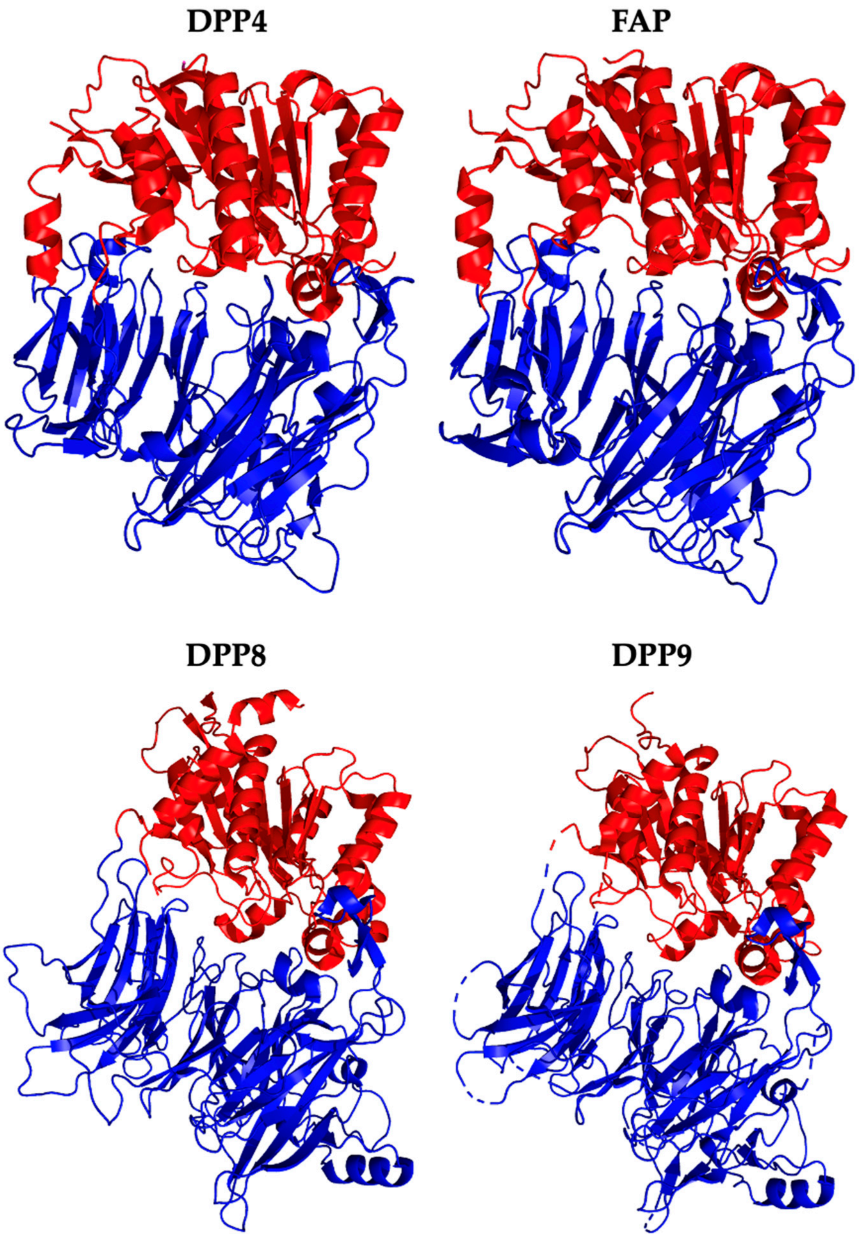

B

```
DPP8            MWKRSEQMKIKSGKCNMAAAMETEQLGVEIFET---ADCEENIESQDRPKLEPFYVERYS      57
DPP9            -MRKVKKLRLDKENTGSWRSFSLNSEGAERMATTGTPTADRGDAAATDDPAARFQVQKHS      59
DPP4            --------------------------------------------------------MKTP      4
FAP             --------------------------------------------------------MKTW      4
                                                                         :

DPP8            WSQLKKLLADTRKYHGYMMAKAPHDFMFVKRNDPDGPHSDRIYYLAMSGENRENTLFYSE      117
DPP9            WDGLRSIIHGSRKYSGLIVNKAPHDFQFVQKTDESGPHSHRLYYLGMPYGSRENSLLYSE      119
DPP4            WKVLLGLL-GAAALVTIITV----PVVLLNKGNDATADSRKTY-------------TLTD      46
FAP             VKIVFGVA-TSAVLALLVMC----IVLRPSRVHNSEENTMRAL-------------TLKD      46
                 . :  :   :      :       .   .: .    .: :                 .:

DPP8            IPKTINRAAVLMLSWKPLLDLFQATLDY--GMYSR---EEELLRERKRIGTVGIASYDYH      172
DPP9            IPKKVRKEALLLLSWKQMLDHFQATPHH--GVYSR---EEELLRERKRLGVFGITSYDFH      174
DPP4            YLKNTYRLKLYSLRWISDHEYLYKQ-ENNILVFNAEYGNSSVFLENSTFDEFGHSINDYS      105
FAP             ILNGTFSYKTFFPNWISGQEYLHQSADNNIVLYNIETGQSYTILSNRTMKSV--NASNYG      104
                  :           *    : :    .    ::.    :.  : ..  :  .     ::

DPP8            -QGSGTFLF-------------QAGSGIYHVKDGGPQGFTQQPLRPNLVETSCPNIRMDP      218
DPP9            -SESGLFLF-------------QASNSLFHCRDGGKNGFMVSPMKPLEIKTQCSGPRMDP      220
DPP4            ISPDGQFILLEYNYVKQWRHSYTASYDIYDL---NKRQLITEERIPNNTQWVTWSPV---      159
FAP             LSPDRQFVYLESDYSKLWRYSYTATYYIYDL---SNGEFVRGNELPRPIQYLCWSPV---      158
                 . .  *:               *   ::.    .   :      *   :    .

DPP8            KLCPADPDWIAFIHSNDIWISNIVTREERRLTYVHNELANMEEDARSAGVATFVLQE-EF      277
DPP9            KICPADPAFFSFINNSDLWVANIETGEERRLTFCHQGLSNVLDDPKSAGVATFVIQE-EF      279
DPP4            ------GHKLAYVWNNDIYVKIEPNLPSYRITWTG------KEDIIYNGITDWVYEEEVF      207
FAP             ------GSKLAYVYQNNIYLKQRPGDPPFQITFNG------RENKIFNGIPDWVYEEEML      206
                         :::: ..::::         ::*:         ::    *:  :* :*  :

DPP8            DRYSGYWWCPKAETTP-SGGKILRILYEENDESEVEIIHV---TSPMLETRRADSFRYPK      333
DPP9            DRFTGYWWCPTASWEGSEGLKTLRILYEEVDESEVEVIHV---PSPALEERKTDSYRYPR      336
DPP4            SAYSALWWSPNGTFLA----------YAQFNDTEVPLIEYSFYSDESLQYPKTVRVPYPK      257
FAP             ATKYALWWSPNGKFLA----------YAEFNDTDIPVIAYSYYGDE--QYPRTINIPYPK      254
                    . **.*..              * : ::::: :*      .   :  ::    **:

DPP8            TGTANPKVTFKMSEIMIDAEGRIIDVIDKELIQPFEILFEGVEYIARAGWTPEGKYAWSI      393
DPP9            TGSKNPKIALKLAEFQTDSQGKIVSTQEKELVQPFSSLFPKVEYIARAGWTRDGKYAWAM      396
DPP4            AGAVNPTVKFFVVNT--DSLSSVTNATSIQITAPAS-MLIGDHYLCDVTWATQERI----      310
FAP             AGAKNPVVRIFIIDT--TYPAYVG---PQEVPVPAM-IASSDYYFSWLTWVTDERV----      304
                :*: ** : : : :      . :      ::  *   :     *:.   *. : :

DPP8            LLDRSQTRLQIVLISPELFIPVEDDVMERQRLIESVPDSVTPLIIYEETTDIWINIHDIF      453
DPP9            FLDRPQQWLQLVLLPPALFIPSTENEEQRLASARAVPRNVQPYVVYEEVTNVWINVHDIF      456
DPP4            ----SLQWLRRIQNYSVMDICDYDESSGRWNCL-------VARQHIEMSTTGWVGR---F      356
FAP             ----CLQWLKRVQNVSVLSICDFREDWQTWDCP-------KTQEHIEESRTGWAGG---F      350
                        *: :     : *    :                     *     * .    *

DPP8            HVFPQSHE-EEIEFIFASECKTGFRHLYKITSILKESKYKRSSGGLPAPSDFKCPIKEEI      512
DPP9            YPFPQSEGEDELCFLRANECKTGFCHLYKVTAVLKSQGYDWSEPFSPGEDEFKCPIKEEI      516
DPP4            RPSEPHFTLDGNSFYKIISNEEGYRHI----------------------CYFQIDKKDCT      394
FAP             FVSTPVFSYDAISYYKIFSDKDGYKHI----------------------HYIKDTVENAI      388
                         :   :    . : *: *:                        ::   ::

DPP8            AITSGEWEVLGRHGSNIQVDEVRRLVYFE-GTKDSPLEHHLYVVSYVNPGEVTRLTDRGY      571
DPP9            ALTSGEWEVLARHGSKIWVNEETKLVYFQ-GTKDTPLEHHLYVVSYEAAGEIVRLTTPGF      575
DPP4            FITKGTWEVIGIEALT-----SDYLYYISNEYKGMPGGRNLYKIQLSDYTK-------VT      442
FAP             QITSGKWEAINIFRVT-----QDSLFYSSNEFEEYPGRRNIYRISIGSYPPS------KK      437
                 :*.* **.:     .        * * .   :  *  :::* :.

DPP8            SHSCCI-SQHCDFFISKYS-------NQKNPHCVSLYKLSSPEDDPTCKTKEFWATILDS      623
DPP9            SHSCSM-SQNFDMFVSHYS-------SVSTPPCVHVYKLSGPDDDPLHKQPRFWASMMEA      627
DPP4            CLSCELNPERCQYYSVSFSKEAKYYQLRCSGPGLPLYTLHSSVNDKGLRVLEDNS-ALDK      501
FAP             CVTCHLRKERCQYYTASFSDYDKYYALVCYGPGIPISTLHDGRTDQEIKILEENK-ELEN      496
                . :* :  :. : :   :*              : : .* .   *   :  .     ::

DPP8            AGPLPDYTPPEIFSFESTTGFTLYGMLYKPHDLQPGKKYPTVLFIYGGPQVQLVNNRFKG      683
DPP9            ASCPPDYVPPEIFHFHTRSDVRLYGMIYKPHALQPGKKHPTVLFVYGGPQVQLVNNSFKG      687
DPP4            ML-QNVQMPSKKLDFIILNETKFWYQMILPPHFDKSKKYPLLLDVYAGPCSQKADTVFRL      560
FAP             AL-KNIQLPKEEIKKLEVDEITLWYKMILPPQFDRSKKYPLLIQVYGGPCSQSVRSVFAV      555
                        * : :         ::  :  *  :: .**:* :: :*.**  * . . *

DPP8            VKYFRLNTLASLGYVVVVIDNRGSCHRGLKFEGAFKYKMGQIEIDDQVEGLQYLASRYDF      743
DPP9            IKYLRLNTLASLGYAVVVIDGRGSCQRGLRFEGALKNQMGQVEIEDQVEGLQFVAEKYGF      747
DPP4            NWATY--LASTENIIVASFDGRGSGYQGDKIMHAINRRLGTFEVEDQIEAARQF-SKMGF      617
FAP             NWISY--LASKEGMVIALVDGRGTAFQGDKLLYAVYRKLGVYEVEDQITAVRKF-IEMGF      612
                         :. .  :. .*.**:  :* ::  *.  ::*  *::**: . : .  ..*

DPP8            IDLDRVGIHGWSYGGYLSLMALMQRSDIFRVAIAGAPVTLWIFYDTGYTERYMGHPDQ--      801
DPP9            IDLSRVAIHGWSYGGFLSLMGLIHKPQVFKVAIAGAPVTVWMAYDTGYTERYMDVPEN--      805
DPP4            VDNKRIAIWGWSYGGYVTSMVLGSGSGVFKCGIAVAPVSRWEYYDSVYTERYMGLPTPED      677
FAP             IDEKRIAIWGWSYGGYVSSLALASGTGLFKCGIAVAPVSSWEYYASVYTERFMGLPTKDD      672
                :* .*:.* ******::: : *     :*: .** ***: *  * : ****:*. *

DPP8            NEQGYYLGSVAMQAEKFPSEPNRLLLLHGFLDENVHFAHTSILLSFLVRAGKPYDLQIYP      861
DPP9            NQHGYEAGSVALHVEKLPNEPNRLLILHGFLDENVHFFHTNFLVSQLIRAGKPYQLQIYP      865
DPP4            NLDHYRNSTVMSRAENFKQ--VEYLLIHGTADDNVHFQQSAQISKALVDVGVDFQAMWYT      735
FAP             NLEHYKNSTVMARAEYFRN--VDYLLIHGTADDNVHFQNSAQIAKALVNAQVDFQAMWYS      730
                * . *  .:*  :.* : .     *::**  *:**** ::  : . *: .   ::   *

DPP8            QERHSIRVPESGEHYELHLLHYLQENLGSRIAALKVI    898
DPP9            NERHSIRCPESGEHYEVTLLHFLQEYL----------    892
DPP4            DEDHGIASSTAHQHIYTHMSHFIKQCFSLP-------    765
FAP             DQNHGLSGL-STNHLYTHMTHFLKQCFSLSD------    760
                :: *.:    : :*   : *:::: :
```

Supplementary Figure 1. Crystal structure and protein sequence of the four enzymatic members in the DPP4 gene family. **(A)** Crystal structure of DPP4, FAP, DPP8 and DPP9. Structures were modified and prepared using PyMOL (Version 2.4.2). **(B)** Multiple-sequence alignment of DPP4 (P27487), FAP (Q12884), DPP8 (Q6V1X1) and the long form of DPP9 (Q86TI2-2). Protein sequences were download from UniProt Consortium and alignment was created using Clustal Omega. . = similar; : = highly similar; * = identical.

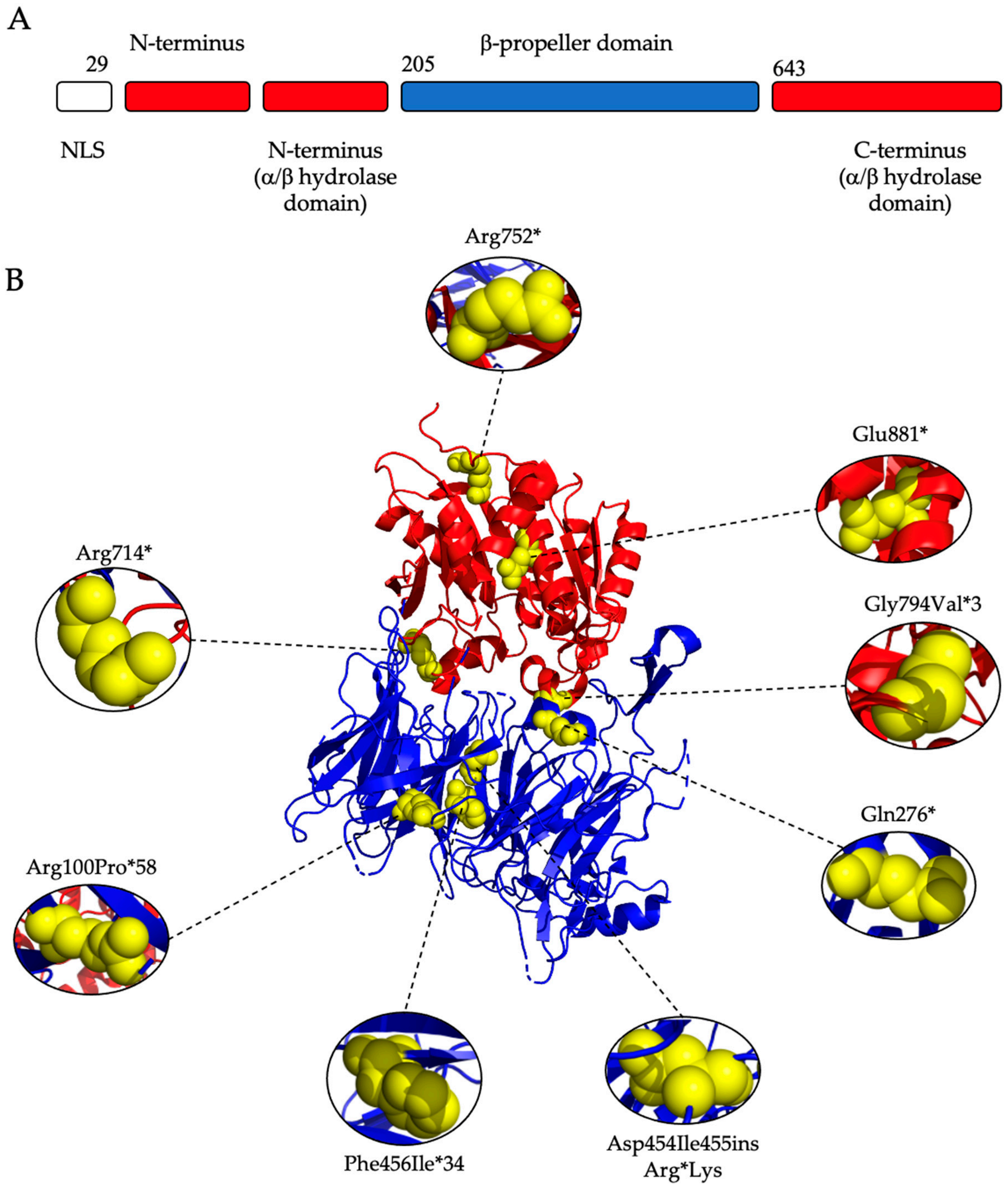
A
N-terminus
β-propeller domain
29
205
643
NLS
N-terminus
(α/β hydrolase
domain)
C-terminus
(α/β hydrolase
domain)
B
Arg752*
Glu881*
Arg714*
Gly794Val*3
Gln276*
Arg100Pro*58
Phe456Ile*34
Asp454Ile455ins
Arg*Lys

C

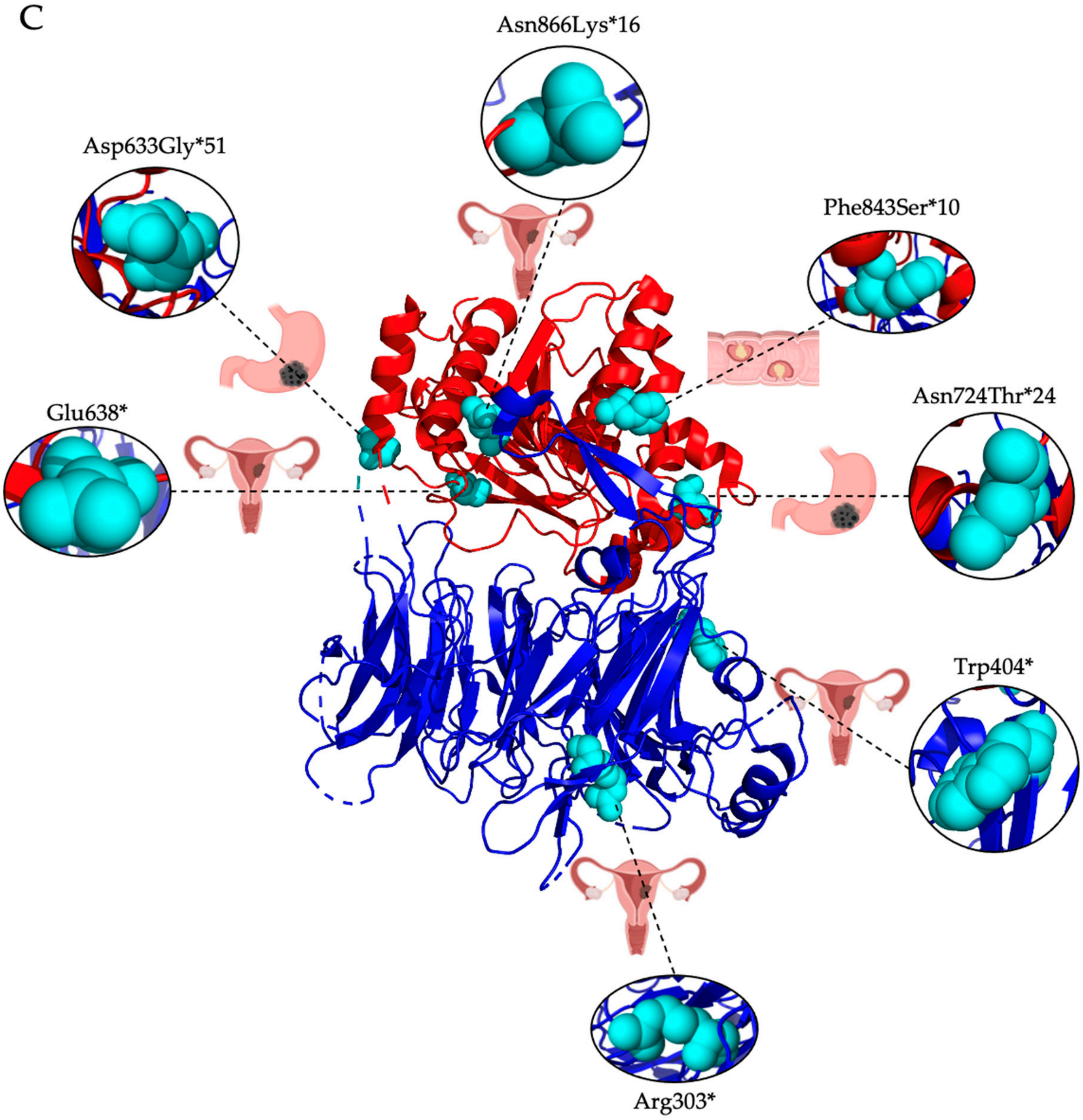
Asn866Lys*16
Asp633Gly*51
Phe843Ser*10
Glu638*
Asn724Thr*24
Trp404*
Arg303*

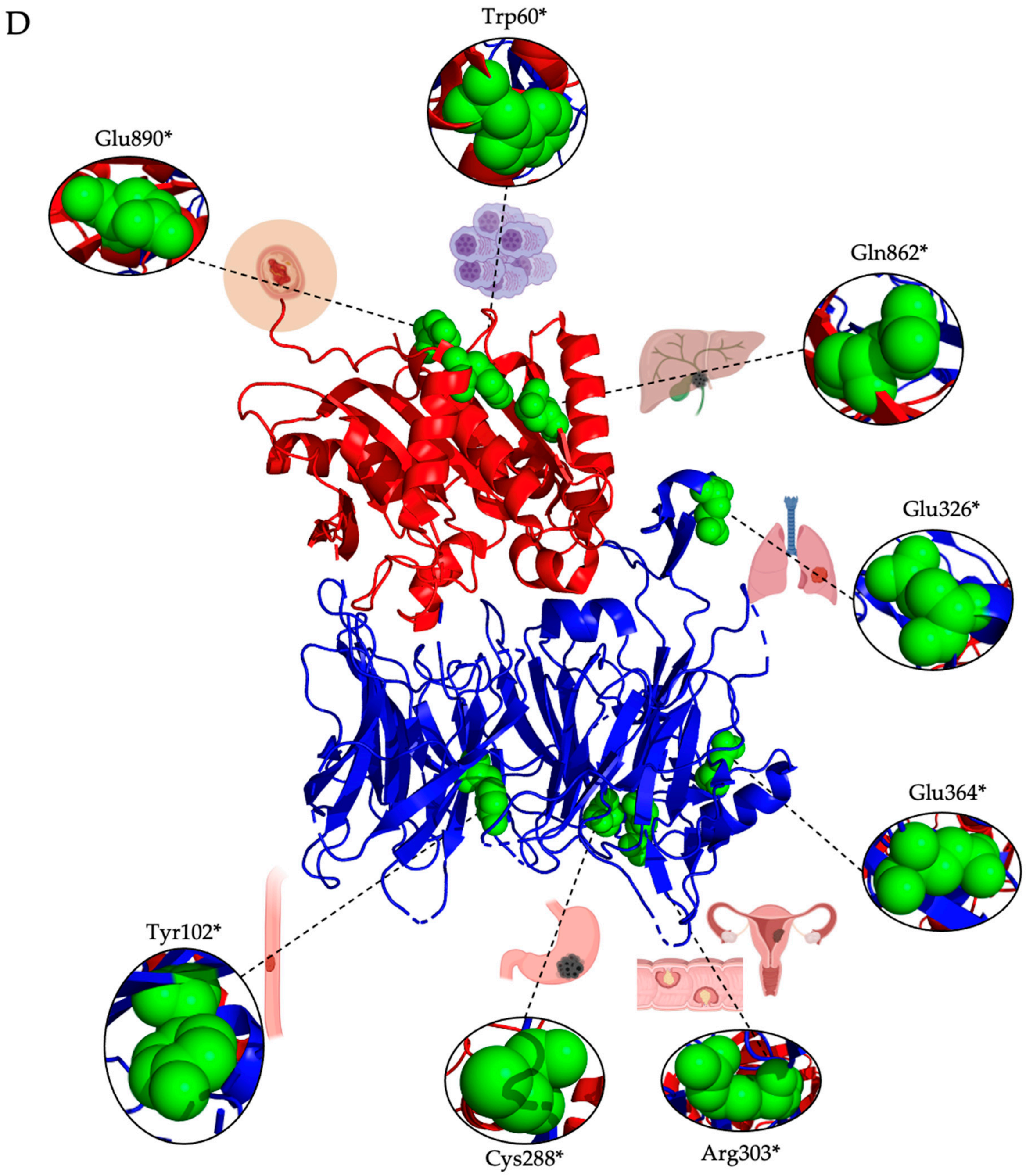

Supplementary Figure 2. *DPP9* variants mapped to the DPP9 protein structure. **(A)** DPP9 protein domain organization. Exonic variants were exported from **(B)** gnomAD, **(C)** TCGA and **(D)** COSMIC databases. The PDB for DPP9 apo structure used here is 6EOQ. The figures were created using PyMOL (Version 2.4.2, Schrödinger, LLC) and BioRender.com. ins = insertion. * Premature termination codon. NLS = nuclear localisation sequence.

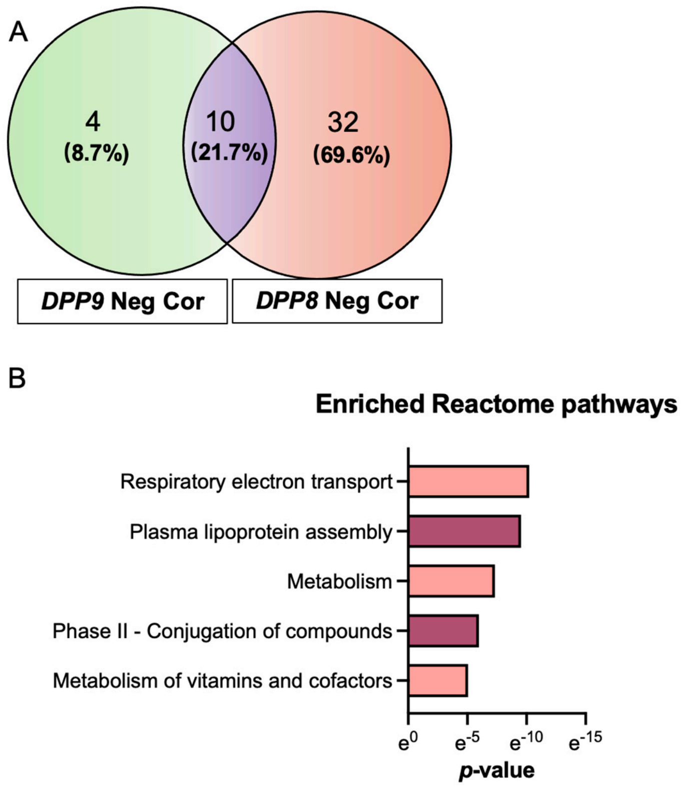

Supplementary Figure 3. Enriched Reactome pathways of genes that were negatively correlated and in-common between *DPP9* and *DPP8*. **(A)** Venn diagram of the genes negatively correlated with *DPP9* and *DPP8*. "Neg Cor" refers to negative correlation. **(B)** Enriched Reactome pathways associated with negatively correlated genes in-common between *DPP9* and *DPP8*. This analysis was performed in ConsensusPathDB, where statistical significance, shown as p value, was calculated hypergeometrically based on the number of entities in the predefined set and the 10 negatively correlated genes defined in the Venn diagram.

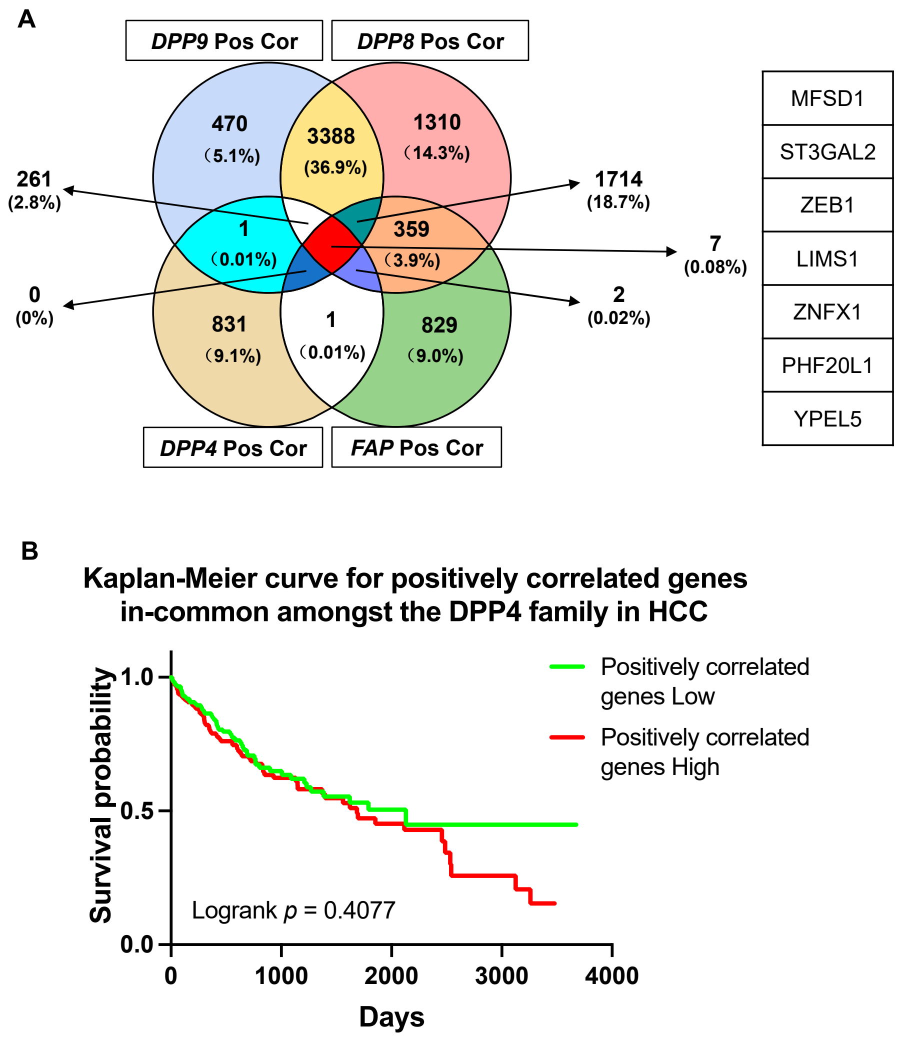

Supplementary Figure 4. Survival analysis on genes that were positively correlated and in-common amongst *DPP9*, *DPP8*, *DPP4* and *FAP* in HCC. **(A)** Venn diagram showing the numbers of genes that were positively correlated in-common with *DPP9*, *DPP8*, *DPP4* and *FAP*. The 7 genes in-common genes with all four genes are listed to the right-hand side. The genes in the blue section and pink section are *TPRA1* and *SH3BP5* respectively. The two genes on the purple section are *VAMP3* and *TTL*. "Pos Cor" refers to positive correlation. **(B)** Kaplan-Meier curve for the 7 genes that were positively correlated in-common amongst *DPP9*, *DPP8*, *DPP4* and *FAP* in HCC patients. The high (red) and low (green) mRNA expression levels of genes in liver tumours were stratified based on median expression value. *p* values were calculated by logrank (Mantel-Cox) test.
